# Image-Guided High-Dose-Rate (HDR) Boost Localization Using MRI/MR Spectroscopy: A Correlation Study with Biopsy

**DOI:** 10.7759/cureus.795

**Published:** 2016-09-21

**Authors:** Eric Vigneault, Khaly Mbodji, Louis G Racine, Eric Chevrette, Marie C Lavallee, André-Guy Martin, Philippe Despres, Luc Beaulieu

**Affiliations:** 1 Radiation-Oncology, Hotel Dieu de Quebc; 2 Centre de recherche du CHU de Québec, CHU de Québec – Université Laval; 3 Département d’imagerie médicale, CHU de Québec – Université Laval; 4 Département de radio-oncologie, CHU de Québec – Université Laval; 5 Département de radio-oncologie, Département de physique, de génie physique et d’optique, CHU de Québec – Université Laval

**Keywords:** prostate cancer, magnetic resonance imaging, magnetic resonance spectroscopy, biopsy

## Abstract

Purpose: The purpose of this study is to compare the blind interpretations of magnetic resonance imaging (MRI) sequences, diffusion-weighted imaging (DWI), apparent diffusion coefficient (ADC), mapping, and magnetic resonance spectroscopy (MRS) of the prostate, in comparison to prostate biopsy to identify a valid dominant intraprostatic lesion (DIL) for dose escalation using high-dose rate brachytherapy.

Methods: MRI/MRS were performed on 20 patients with intermediate risk adenocarcinoma of the prostate. T1W, T2W, DWI-ADC, and MRS sequences were performed at 1.5 T with pelvic and endorectal coils. An experienced radiologist rated the presence of cancer in each sextant by using a dichotomic approach, first on MR standard acquisitions (T1W and T2W), then on DWI-ADC mapping, and later on MRS images. Areas under the receiver’s operating characteristic curve were calculated using a sextant as the unit of analysis. The transrectal ultrasonography-guided biopsy results were used as the reference standard. A table summarizing the MRI/MRS findings was made and compared to the corresponding area in the prostate biopsy report. A perfect match was defined to be the presence of cancer in the same sextant of the MRI/MRS exam and the prostate biopsy.

Results: The interpretation of the MRI/MRS exams per sextant was compared to the diagnostic biopsy report. MRI readings were compared with the biopsy as a surrogate for the complete pathology specimen of the prostate. A sensitivity (Sn) of 98.6% (95% confidence interval, 92.2% - 99.9%) and specificity (Sp) of 60.8% (46.1% - 74.2%) were found. The positive and negative predictive values (PPV, NPV) were 77.3% (67.1% - 85.5%) and 96.9% (83.8% - 99.9%), respectively. When MRS readings were compared with biopsy, we found a Sn of 96.4% (87.7% - 99.6%) and Sp of 54.8% (38.7% - 70.2%). The PPV and NPV were 74% (62.4% - 83.6%) and 92% (74% - 99%), respectively. DWI-ADC mapping results were also compared with biopsy. We found a Sn and Sp of 93.7% (84.5% - 98.2%) and 82.1% (66.5% - 92.5%), respectively, and a PPV and NPV of 89.4% (79.4% - 95.6%) and 88.9% (73.9% - 96.9%), respectively. Finally, after combining MRI, MRS, and DWI-ADC mapping, compared with biopsy, we obtained a Sn, Sp, PPV, and NPV of 100% (94.8% - 100%), 49% (34.8% - 63.4%), 72.6% (62.5% - 81.3%), and 100% (86.3% - 100%), respectively.

Conclusions: The combination of MRI/MRS is a sensitive tool for both the structural and metabolic evaluation of prostate cancer location. MRI/MRS exams are useful to delineate a DIL for high-dose-rate (HDR) intraprostatic boost.

## Introduction

Prostate cancer is the most common cancer in Canadian men. In 2015, an estimated 100,500 new cases of cancer in males were expected to be diagnosed in Canada. Among them, prostate cancer represented the leading type of cancer with 24,000 new cases [1]. About a third of prostate cancers diagnosed are classified as an intermediate-risk disease based on their clinical features [2]. The incidence of prostate cancer has been on the rise since 1980, but it is now stabilizing [1]. Due to the widespread availability of serum prostate specific antigen (PSA), the majority of patients are diagnosed with an organ-confined disease. However, it is known that the vast majority of prostate cancers are multifocal [3]. Historically, radical radiotherapy was aimed at treating the entire gland rather than individual cancer foci. The outcome of treatment for localized prostate cancer with radiotherapy has improved considerably in the last 25 years [1]. Even today, typical radiation treatment encompasses the entire prostate without specifically targeting cancerous areas. The increased precision and conformity of external beam and brachytherapy modalities have led to dose escalation protocols that maintain low toxicity and greatly impact local tumor control rates [4-6]. Randomized trials have shown that escalation of radiotherapy doses improves biochemical disease-free survival in prostate cancer patients [7]. For intermediate-risk and high-risk patients, studies have demonstrated a benefit of higher doses [6]. Patients in whom local control is achieved are also less likely to develop distant metastases [7]. Dose escalation can be accomplished by increasing either the absolute dose of radiation or the biological equivalent dose [8]. High-dose-rate (HDR) brachytherapy is an excellent way to attain highly conformal high-dose-per-fraction radiation to the prostate. This hypofractionation scheme has been used to enhance tumor control and reduce early sequelae [5].

Traditional methods for evaluating prostate cancer, such as digital rectal examination, PSA level, and transrectal ultrasonography, do not allow the localization of malignant foci in the prostate. Ultrasound images provide excellent guidance to the physician regarding the gland size and its boundaries but yield limited information on internal glandular tissue and little to no detail on focal lesions [9]. MRI provides the best depiction of the prostate internal zonal anatomy. In addition, MRI also allows functional assessment with sequences, such as DWI-ADC mapping and MRS. Traditionally, MRI has been used for the locoregional staging of disease in men with biopsy-proven cancer [10]. Functional imaging modalities, such as combined MRI/MRS, provide new tools to better detect tumor foci. The use of T2W imaging revealed accuracies of 67% - 72% in tumor localization [11-12]. The addition of MRS imaging resulted in a 90% positive predictive value for localization of tumors in the peripheral zone of the prostate. MRS of prostate cancer tissues shows an increased choline level and a reduced citrate level [13]. The use of DWI-ADC mapping, in addition to T2W imaging, showed an improvement in detection of prostate cancer in the transition zone [14].

In this context, the purpose of this study was to compare MRI/MRS prostate studies to biopsy results in order to assess the relevance of using these imaging techniques before the planning of radiotherapy treatments. A good detection and localization of lesions with MRI/MRS could improve the treatment by selecting a valid dominant intraprostatic lesion for dose escalation with an HDR boost in addition to external beam radiotherapy (EBRT).

## Materials and methods

The Ethical Committee of CHU de Québec approved this study, and all patients provided their written informed consent. Upon consultation for management options regarding their prostate cancer, patients were stratified into risk groups based on clinical and pathological parameters as detailed in the guidelines of the National Comprehensive Cancer Network (NCCN) [15]. Inclusion criteria were intermediate-risk prostate cancer (PSA between 10 and 20 ng/ml, clinical stage T2B or T2C, or Gleason score 7), available histopathological topographic prostate biopsy map, and no contraindication for an MR examination of the prostate.

Each patient underwent a prostate transrectal ultrasonography-guided biopsy. Multiple specimens were taken in order to map the entire gland. The transrectal ultrasonography biopsy was the reference standard for the 20 patients as a surrogate for the complete pathology specimen of the prostate gland; six to 20 biopsies were obtained from each patient. At least eight weeks after the biopsy, patients were subject to an MR study. All MR exams included T1W, T2W, and spectroscopy acquisitions; 17 exams also included a DWI sequence with ADC mapping. The entire prostate and seminal vesicles were imaged in each patient. The biopsies were performed before the MRI/MRS by the referring urologist. At the time of the referral to radiation oncology, if the patient consented to participate in the study, the MRI was performed and interpreted blindly by an independent observer according to the same sextant anatomy that is used for the biopsy. A Prostate Coil-BPX Series (disposable endorectal coils-MEDRAD) (Bayer Healthcare LLC, Whippany, NJ) was used for acquisition with a GE 1.5 T MR (GE Healthcare, Milwaukee, WI). The reconstructed slice thickness was 3-4 mm, depending on the sequence. The duration of the scan was approximately 25 minutes.

### MRI/MRS protocol

For image analysis and tumor localization, each prostate was subdivided into the base, mid-gland, and apex regions. Each of these subdivisions was defined as being right or left according to a sagittal midplane line. In each case, six regions of interest (ROI), called sextants, were defined [16].

One radiologist reviewed all of the MR imaging studies. The radiologist was aware that all patients had biopsy-proven prostate cancer but did not know the localization of the tumors in the gland. MRI standard sequences (T1W and T2W), DWI, and MRS were analyzed independently. For each sextant, the reader marked the ROI as positive if cancer was found in the subdivision or negative if no cancer was found. Some sextants were graded as noninterpretable if the image quality was insufficient for interpretation.

T1W and T2W images were first evaluated. The criterion for assessing prostate cancer was a low-intensity signal on the T1W image, corresponding to a low-intensity signal region on the T2W image. Findings had to be consistent with each other in order to define a sextant as positive for cancer [17]. Then, DWI-ADC mappings were evaluated. Criteria for determining a positive sextant were areas of diffusion restriction and a hyperintense lesion in DWI and/or hypointense lesion in ADC mapping, relative to the surrounding gland [18].

Finally, MRS sequences were evaluated. Peaks of choline (Cho), citrate (Cit), and creatine (Cre) were evaluated. In a healthy prostate, the Cho/Cit ratio is low. However, in prostate cancer, Cit concentration decreases and Cho concentration increases; the concentration of Cre remains relatively stable. Each voxel where the Cho+Cre / Cit was higher or equal to 0.70 was considered as positive for cancer [19]. Once one positive voxel was found in a sextant, the whole sextant was marked as positive.

Each sextant (n = 173) was considered independently and then was compared to biopsy results. Sensibility (Sn), specificity (Sp), positive predictive value (PPV), and negative predictive value (NPV) were calculated with a confidence interval of 95%. Each sequence (MRI standard acquisitions (T1W and T2W), DWI-ADC mapping, and MRS) was compared to histopathology results. Finally, T1W/T2W, MRS, and DWI-ADC results were all combined together, and the combination was compared to histopathology results. In the case of combined acquisitions, prostate cancer was defined and scored according to the Prostate Imaging - Reporting and Data System (PI-RADS). We found that in two, seven, and 11 patients, the prostate cancer identified was of type 3, 4, and 5, respectively, according to the new PI-RADS: 2015, Version 2 (PI-RADS™ v2) [20]. All statistical analyses were performed using SPSS software, version 23.0 (IBM Corporation, Armonk, NY USA). 

## Results

Between December 2009 and March 2011, 20 patients (mean age: 64 years; range: 53-80 years) were recruited for this study. Gleason score was 7 for all patients, and pretreatment PSA levels ranged between 0.31 and 9.70 ng/ml with a mean of 5.4 ng/ml. All patients underwent a clinical rectal examination, routine blood tests, and routine staging studies to exclude metastatic disease. Patient characteristics, including age, pretreatment PSA levels, T stages, and biopsies, are summarized in Table 1.

**Table 1:** Demographic Characteristics of Patients (n = 20) SD = standard deviation; PSA = prostate specific antigen

Age (Years)	
Mean (SD)	64.2 (7.3)
Range	(53-80)
Stage	
T1 (n, %)	12 (60%)
T2 (n, %)	8 (40%)
PSA Pre-Tx (ng/ml)	
Mean (SD)	5.4 ± 2.6
No. biopsies/patient	
Mean (SD)	7 (4)
No. positive biopsies/patient	
Mean (SD)	3 (3)

**Figure 1:**
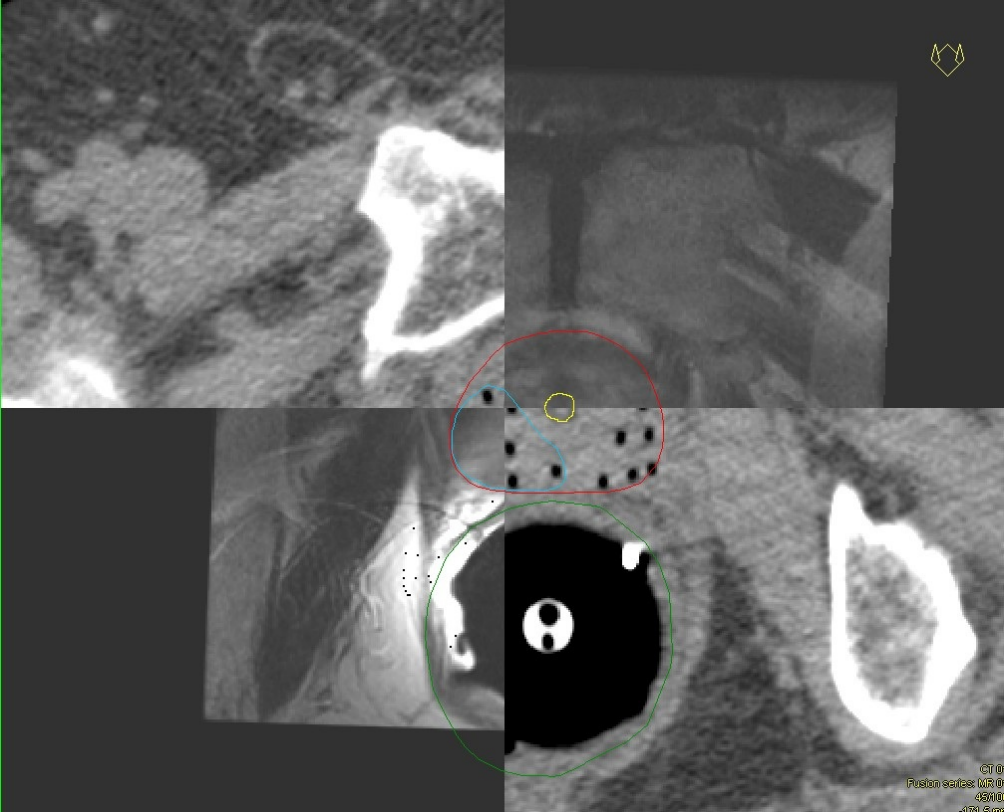
Image fusion between the MRI and the post-implantation CT with catheters to delineate the DIL to boost

Table 2 shows the Sn, Sp, PPV, and NPV for all imaging techniques. For the MRI standard sequences (T1W and T2W), the radiologist predicted 88 malignant sextants out of 120. With regard to biopsy results, Sn was 98.6% and Sp 60.8% with 95% CI of 92.2% - 99.9% and 46.15 - 74.2%, respectively. PPV was 77.3% and NPV 96.9% with 95% CI of 67.1% - 85.5% and 83.8% - 99.9%, respectively. Figure 1 presents an image fusion between the MRI and the post-implantation computed tomography (CT) with catheters to delineate the DIL for HDR boost.

**Table 2:** Sensitivity (Sn), specificity (Sp), positive predictive value (PPV) and negative predictive value (NPV) of MRI, MRS, DWC-ADC and combined MRI/MRS/DWI-ADC for cancer detection compared to biopsies used

	MRI	MRS	DWI-ADC	MRI/MRS/DWI-ADC
Sn (%, 95% CI)	98.6 (92.2-99.9)	96.4 (87.7-99.6)	93.7 (84.5-98.2)	100 (94.8-100)
Sp (%, 95% CI)	60.8 (46.1-74.2)	54.8 (38.7-70.2)	82.1 (66.5-92.5)	49 (34.8-63.4)
PPV (%, 95% CI)	77.3 (67.1-85.5)	74 (62.4-83.6)	89.4 (79.4-95.6)	72.6 (62.5-81.3)
NPV (%, 95% CI)	96.9 (83.8-99.9)	92 (74-99)	88.9 (73.9-96.9)	100 (86.3-100)
No. of sextants	120	102	98	120
No. of sextants positive (%)	88 (73%)	66 (65%)	73 (74%)	95 (79%)

For the DWI-ADC mapping, only 102 sextants were available for analysis instead of 173. DWI-ADC mapping was not available for the first three patients out of 20 and they did not have that sequence included in their MR examination. The reader declared 66 positive sextants. In comparison to histopathological results, Sn, Sp, PPV, and NPV were calculated to be 93.7%, 82.1%, 89.4%, and 88.9% with a 95% CI of 84.5% - 98.2%, 66.5% - 92.5%, 79.4% - 95.6%, and 73.9% - 96.9%, respectively.

Regarding MRS, the radiologist found there to be Cho + Cre/Cit ratio inversion in 73 sextants out of a maximum possibility of 98. Twenty-two sextants were not interpretable and were excluded from the analysis. The impossibility to read those voxels was mainly caused by the presence of residual blood after the insertion of fiducial markers. We found a Sn of 96.4%, Sp of 54.8%, PPV of 74%, and NPV of 92% with 95% CI of 87.7% - 99.6%, 38.7% - 70.2%, 62.4% - 83.6%, and 74% - 99%, respectively.

Finally, all three sequences were combined together (T1W and T2W, DWI-ADC mapping, and MRS). Ninety-five sextants out of 120 were considered positive. For these 95 sextants, at least one sequence out of three was positive for the ROI. We obtained 100% Sn and 49% Sp with 95% CI of 94.8% - 100% and 34.8% - 63.4%, respectively; PPV and NPV were 72.6% and 100% with a 95% CI of 62.5% -81.3% and 86.3% - 100%, respectively.

In this research, every sequence was useful for detecting cancer foci in the prostate. We obtained a perfect match with the combination of MRI, MRS, and DWI-ADC mapping while MRI standard acquisitions, MRS, and DWI-ADC mapping alone showed a performance of more than 90% each in detecting cancer. The negative predictive values of each sequence and combined sequences also showed interesting results. On the other hand, Sp results were lower than expected, varying between 49% and 82.1%.

## Discussion

There is evidence that prostate cancer most often recurs in previously affected areas [21]. There is also evidence of the benefit of dose escalation, especially in intermediate risk prostate cancer [22]. Thus, increasing the dose to involved areas while treating the entire prostate gland to standard dose appears to be an appealing way to improve the therapeutic ratio. Brachytherapy, designed and delivered with accurate anatomic reference, is well-suited for dose escalation. By virtue of the inverse square law, brachytherapy results in a much steeper dose gradient and, hence, can achieve an improved therapeutic ratio compared to external beam radiotherapy [23].

According to the PI-RADS v2, the dominant intraprostatic lesion (DIL) is defined by the volume of suspicious cancer assessed by MRI/MRS in a zone where the biopsy was positive. In this study, we were not targeting microfoci of cancer for the HDR boost but rather the high cancer volume area, which corresponds to the DIL. In order to identify the DIL, the combination of MRI and MRS has already shown high sensitivity and specificity when compared to the histopathological specimen following radical prostatectomy. Several papers in the literature have shown high Sn and Sp ranges. Namely, Aydin, Futterer, and Testa are among the authors that have studied MRI/MRS Sn and Sp in prostate cancer detection [24-26]. In these studies, the authors obtained good Sn and Sp values in MR spectroscopy alone or combined with MR imaging and diffusion-weighted imaging apparent diffusion coefficient mapping sequences. Due to its high sensitivity and specificity, MRI with MRS was used to select the boost target DIL, as previously demonstrated by the UCSF group [27].

In the absence of the entire prostate gland histopathological specimen, sextant biopsy findings were relied upon for localization of cancer foci within the prostate. In this study, the objective was to evaluate the correlation between the prostate biopsy and the MRI, DWI-ADC mapping, and MRS in order to define a boost target DIL for HDR. The above-mentioned study is not the first to compare prostate biopsy to MRI/MRS results, but it was the first clinical study to use MRI/MRS to define DILs to treat patients with an HDR brachytherapy boost. Figure 1 illustrates image fusion between the MRI and the post-implantation CT with catheters to delineate the DIL to boost. This study is the first step towards image-guided brachytherapy boost. Using automatic shimming for MRS, we obtained promising results for the definition of DILs. Indeed, in each sequence, the results showed excellent values for Sn, PPV, and NPV, but low values were obtained for specificity (Sp). When the different analyses were combined, we observed a Sn and NPV of 100%, while the Sp and the PPV were lower compared to the results obtained in each analysis. This decrease in the Sp could probably be explained by the large number of false positive readings. Indeed, disease progression between the time of the biopsy and the MRI/MRS study, or the geographic miss of the biopsy may explain the more extensive disease found in the case of MRI/MRS. As previously reported by Wefer, et al., biopsy alone is a limited method for localizing prostate cancer. The tumor occupies only a part of each sextant and core biopsy samples; only performing the biopsy in a small area of each sextant could easily result in missing the tumor [16].

In some studies, MRI/MRS readings were compared to prostatectomy specimens [28-29]. However, patients who opted for an organ conservative treatment, such as external beam radiotherapy or brachytherapy, did not get their prostate removed for analysis. Consequently, a descriptive histopathology report of the prostate biopsy was the only source of information. Also, low T2 signal intensity in the peripheral zone is the primary MRI sign of tumor presence, but it may also be caused by post-biopsy hemorrhage and prostatitis. One solution to avoid the time interval between biopsy and MRI/MRS, as well as the artifact from bleeding after biopsy, would be to perform the MRI/MRS shortly before a repeated biopsy. Furthermore, in order to increase the correlation between MRI and MRS, a minimum of 12 core biopsies should be performed.

We hypothesized that information provided by MRI and MRS techniques could significantly improve detection and localization of neoplasm foci in order to define a DIL in patients with intermediate risk prostate cancer. The definition of DILs based on MRI/MRS was used to boost selectively the DIL with HDR brachytherapy and will be reported in another publication.

This study was not without limitations. Firstly, transrectal ultrasonography biopsy was the reference standard for the 20 patients as a surrogate of the complete pathology specimen of the prostate. Biopsies were taken at the periphery of the prostate; some lesions in the paramedian zones could have been missed, especially in patients who had as few as six biopsies. Biopsies were not performed by the same urologic surgeon; nonetheless, this reflects the day-to-day clinical reality. Though most of the biopsies were done in the same hospital center, the few which were not may potentially have increased the variability of the results. Secondly, the sample size is relatively small and, by considering each sextant independently (n = 173) for a population of 20 patients, there is probably an interdependence between the results. Finally, automatic shimming in the MRS study was used. Manual shimming could improve the quality of the spectrum resulting from an MRS study, thus, improving interpretation.

## Conclusions

The combination of MRI/MRS is a sensitive tool for both the structural and metabolic evaluation of prostate cancer location. In this study, the reported specificity is lower than expected but could be improved by repeating the sextant biopsy shortly after the MRI/MRS, if needed. Noteworthy, the information obtained from the combination of all the sequences appear to be the best way to identify the DIL for HDR intraprostatic boost. After a longer follow-up, the clinical outcomes of this cohort of patients will be reported.
